# Population Status of Sympatrically Breeding Skuas (*Catharacta* spp.) at Admiralty Bay, King George Island, Antarctica: A Case Report for 2020–2024

**DOI:** 10.3390/biology14030305

**Published:** 2025-03-17

**Authors:** Katarzyna Komarowska, Katarzyna Fudala, Michał Dziembowski, Alexander Hagge, Robert Józef Bialik

**Affiliations:** 1 Faculty of Biological and Veterinary Sciences, Nicolaus Copernicus University, 87-100 Torun, Poland; 2 Institute of Biochemistry and Biophysics, Polish Academy of Sciences, 02-106 Warsaw, Poland; michal.dziembowski@ibb.waw.pl (M.D.); haggealexander@gmail.com (A.H.)

**Keywords:** Important Bird and Biodiversity Areas, King George Island, ASMA No. 1, Admiralty Bay, south polar skua, brown skua, population fluctuations of seabirds, breeding site IBAs in Antarctica

## Abstract

The present study sets out to examine the status of sympatric populations of south polar (*Catharacta maccormicki*) (SPSs) and brown skuas (*Catharacta antarctica*) (BSs) at two sites on King George Island, Antarctica. The study sites were designated as Important Bird and Biodiversity Areas (IBAs) and were monitored for three (for Point Hennequin (PH)) and four (for West Admiralty Bay (WAB)) consecutive breeding seasons, concluding with the 2023/24 season. The most recently reported data from these areas are from the 2004/05 season, and the data presented herein allow both areas to be assessed in the context of their IBA and Antarctic Specially Protected Area values. The mean number of total pairs for the investigated seasons for WAB was 67 ± 7, while for PH, it was 157 ± 18. The number of pairs of SPSs at PH and WAB justifies the positive evaluation of the areas as IBAs based on global designation criterion A4 proposed jointly by BirdLife International and the Scientific Committee on Antarctic Research (SCAR). PH is of particular interest, as it has been determined that at least 2.3% of the global population of SPSs, estimated by BirdLife International to be between 6000 and 15,000 adult individuals, breeds at this site. The expansion of both IBA boundaries is also recommended based on this study.

## Simple Summary

The present study monitored the populations of south polar and brown skuas in two Important Bird and Biodiversity Areas (IBAs) of Admiralty Bay, King George Island, Antarctica, over the course of the 2020/21 to 2023/24 seasons. The breeding territories of both species were mapped and surveyed regularly from November to March in order to determine the total number of pairs of each species observed in the area, as well as the number of breeding pairs and the level of breeding success. The population data presented can be used to re-evaluate the two Important Bird and Biodiversity Areas, as well as to improve the management mechanisms of Antarctic Specially Protected Area No. 128 (which overlaps with the IBA AQ046). Given the threats to the fragile stability of the Antarctic ecosystem, including climate change and anthropogenic environmental impact, population studies are of great value, particularly of seabirds, which are recognized as the sentinels of global environmental changes.

## 1. Introduction

The brown skua (*Catharacta antarctica*, BS) and the south polar skua (*Catharacta maccormicki*, SPS) are two closely related seabird species from the family Stercorariidae [1]. Inhabiting the Antarctic region, they exhibit distinct ecological adaptations and morphological traits, while sharing key behavioral and life history characteristics, such as high site fidelity, long lifespan, and aggressive territorial behavior, particularly during nesting [2,3,4,5]. Their speciation is considered incomplete, and they are sometimes grouped with the Chilean skua (*Catharacta chilensis*) as a superspecies [6,7]. Further complicating their taxonomy, the BS population examined in this study is occasionally classified as a separate species (*Catharacta lonnbergi*) or as an allopatric breeding subspecies (*Catharacta antarctica lonnbergi*) collectively referred to as the sub-Antarctic skua [6,8]. In contrast, the SPS is not subdivided into distinct subspecies and is generally recognized as a full species [8].

The BS is predominantly found on sub-Antarctic islands and the Antarctic Peninsula; whereas, the SPS breeds at higher latitudes, including the Antarctic continent. Although their breeding distributions and nest site choices generally differ, a 500 km-wide zone of overlap exists, where interbreeding may occur, producing viable and fertile hybrids [9]. This hybrid zone includes King George Island, where mixed-species pairs and hybrid individuals have been observed [5]. Distinguishing hybrids from pure species can be challenging, particularly beyond the first generation [9]; however, most adults of each species can be reliably identified based on size and plumage coloration [4]. The feeding ecology of the adult hybrids are poorly studied [10,11,12] but is presumed to align more closely with the dietary strategy of the sympatric SPS [13]. While the pure BS in sympatry functions as a generalist predator and scavenger, frequently preying on penguin chicks and foraging on marine carrion [11,12], the SPS exhibits a more opportunistic foraging strategy, relying primarily on available marine food resources [11,12,14]. The extent to which hybrids adopt a mixed feeding approach or predominantly follow the SPS pattern remains uncertain.

Despite the SPS having a relatively lower reproductive output compared to the BS [3], both species are listed as “least concern” by BirdLife International. Their estimated global population is thought to be stable and comprises c. 8000 SPS breeding pairs [2,8] and c. 26,000–28,000 mature BS individuals [15]. However, in the study area, an unexpected demographic trend has been observed; between 1978/1979 and 2004/2005, the number of SPS breeding individuals increased by 293%; whereas, the BS population—despite its higher productivity—declined by 40% during the same period [5]. Although the number of mixed and hybrid pairs also increased, it does not fully account for the substantial decline in the more reproductively successful species. This pattern is particularly perplexing given the contrasting riskiness of migratory strategies of the two species. The SPS undertakes long and high-risk transequatorial migrations; whereas, the BS is largely sedentary, with no strong evidence of regular dispersal to the North Atlantic.

In this context, the establishment and maintenance of Antarctic Specially Protected Area No. 128 (ASPA) play a crucial role in minimizing at least the direct anthropogenic impact on the species by restricting human activities in critical breeding and foraging habitats of BSs, thus ensuring the conservation not only of this species but also of other coexistent ecologically significant seabirds [16].

In accordance with the provisions of Annex V of the Madrid Protocol on Environmental Protection to the Antarctic Treaty, the designation of an Antarctic Specially Protected Area (ASPA) is permitted for any area, including a marine area, with the objective of safeguarding environmental, scientific, historical, aesthetic, or wilderness values [17]. The Antarctic Treaty stipulates a list of protected values within the ASPA network; however, it does not provide clear and quantitative methods for identifying and prioritizing such areas. As noted by Harris and Woehler [18], this may result in the selection of sites that comprise the ASPA network being driven by the national interests of countries with scientific or logistical establishments in Antarctica, rather than by efforts to select a representative and standardized network of protected areas.

In the 1990s, BirdLife International and the Scientific Committee on Antarctic Research (SCAR) collaborated to perform the Important Bird Area (IBA) Inventory in the Antarctic, resulting in an initial list of significant bird habitats in the region. Subsequently, the list underwent a secondary review, incorporating new publications and alterations in population status for species, by Environmental Research and Assessment (ERA), on behalf of BirdLife International and the UK Foreign and Commonwealth Office. The final report [19] included 101 IBAs, 40 of which were for the South Shetland Islands (SSI), 14 for King George Island (KGI), and 1 for Admiralty Bay (IBA West Admiralty Bay, King George Island). Four years later, the status of Antarctic IBAs selected in 2011 was updated, and a new, more comprehensive report was published [20]. The total number of IBAs increased to 204, with additional 36 IBAs designated for SSI, 11 for KGI, and 2 for Admiralty Bay, i.e., IBA AQ045, Point Hennequin and IBA AQ046, West Admiralty Bay (henceforth referred to as IBA PH and IBA WAB). According to the BirdLife database [21], all IBAs included in the 2015 report are still considered active. The list includes marine IBAs and two terrestrial IBAs, namely Ryder Bay (BirdLife International, 2025 [22] based on Philips et al. [23]) and Cape Melville (BirdLife International, 2024 [24] based on Fudala and Bialik [25]) that have been added to the list since the report was published.

It is imperative to acknowledge the revisions and updates made by BirdLife International in 2020 [26] to the nomenclature of the criteria employed for the identification of Important Bird and Biodiversity Areas (IBAs). The most substantial of these revisions pertains to the transfer of criteria A4i-A4iv to a unified global IBA criterion A4, which is articulated as follows: “The site is known or thought to hold congregations of ≥1% of the global population of one or more species on a regular or predictable basis”. It should be noted, however, that Antarctic IBAs classified prior to 2020 used the old designation/selection criteria.

As many Antarctic seabird species’ populations were examined for the purposes of creating and implementing the Antarctic IBAs network, there was only one case in which the BS was a trigger species for the establishment of an IBA, i.e., IBA AQ019, Signy Island [20]. Meanwhile, the SPS was a trigger species for the establishment of 24 IBAs, and in 12 cases, it was the only species solely contributing to this criterion being met [4]. IBA PH was classified under criterion A4ii, based on the study by Costa and Alvez [11] describing the results of a bird inventory carried out at Point Hennequin during the 2004/05 season. Within the 277 ha that make up the IBA PH, 116 breeding pairs of SPS were recorded, along with 7 other species of nesting birds. The second IBA belonging to ASMA 1, IBA WAB, has been classified under criteria A4ii and A4iii. The boundaries of IBA WAB coincide with the extent of ASPA No. 128—the area, which has been identified as particularly valuable for birds due to the large number of breeding Gentoo penguins and the concentration of other seabirds [20]. For both investigated IBAs, the latest published data on skua populations are from the 2004/05 season [5,27].

The objective of this study was twofold: firstly, to inventory sympatrically breeding skua species (*Catharacta* spp.) in two IBAs (PH and WAB) and their surrounding ice-free areas over a 4-year period; and secondly, to present the results of inter-seasonal changes in the abundance of both species in these areas. This overarching objective seeks to address the following questions: (1) Does the number of pairs of both species in these areas support the continued IBA status of AQ045 PH and AQ046 WAB? (2) Do the analyzed species meet IBA criterion A4 for AQ046 WAB? (3) Should the boundaries of both IBAs be updated and potentially extended?

## 2. Materials and Methods

### 2.1. Study Area

**Figure 1:**
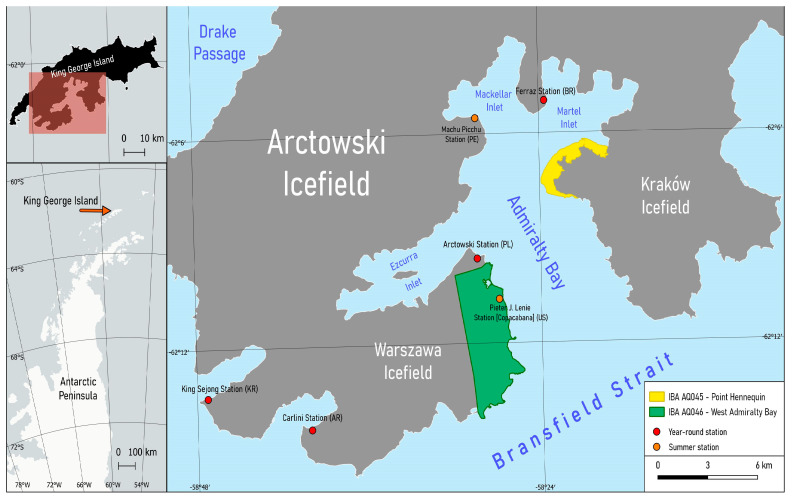
The two study sites adjacent to Admiralty Bay, King George Island, South Shetland Islands, Antarctica.

The inventory of SPS and BS breeding pairs was conducted in two IBAs located in Admiralty Bay, King George Island, Antarctica (Figure 1). In addition, the surrounding areas not covered by ice were also investigated.

IBA AQ045 Point Hennequin (58°22′48″ W, 62°06′57″ S) is situated on the eastern shore of Admiralty Bay, at the entrance of Martel Inlet, covering an area of approximately 277 ha. The IBA PH extends from Warkocz in the northeast to Basalt Point in the south [28]. For this study, only the southern part of IBA, which harbored the highest number of skua pairs, was examined during the seasons of 2021/22 and 2022/23. The whole IBA HP, including Point Hennequin, Smok Hill, and Warkocz, was surveyed during the 2023/24 season. IBA AQ046 West Admiralty Bay (58°28′ W, 62°12′ S) covers an area of 1804 ha and coincides with the boundaries of the Antarctic Specially Protected Area No. 128. Its northern boundary extends to Penguin Ridge, approximately 0.5 km south of the Arctowski Station, while the southernmost point is marked by Patelnia Point [29]. The western linear and north–south-oriented boundary encompasses the ice-free area as well as the eastern parts of Warszawa Icefield. Additionally, the study area included an ice-free region to the north (Penguin Ridge, Jasnorzewski Gardens, Ubocz, Upłaz, and Point Thomas), northwest (Panorama Ridge, Krokiew, Jersak Hills, Wróbel, and Italia Valley), as well as the southwest (Red Hill) of the IBA WAB. For the purpose of this study, this combined area will be referred to as the “outside regions”.

### 2.2. Sampling

The IBAs and the outside regions were regularly visited during four breeding seasons: 2020/21 (this season only applies to IBA WAB), 2021/22, 2022/23, and 2023/24. Each season began when the first skuas were observed establishing territories and ended when the last fledgling left the study areas. The exact start and end dates varied between seasons but generally ranged from November to March, with few observations extending until April. Based on the data on two skua species, BS and SPS, from these seasons, five phenological stages were identified in IBA WAB and three stages in IBA PH (see Table A1, Table A2 and Table A3). The dates for these stages were determined empirically, considering the phenology of the majority of breeding skuas.

Point Hennequin was surveyed at least three times per season to assess the distribution of the skua pairs in the southern part of IBA PH (2021/22 and 2022/23 seasons) or the entire IBA PH (2023/24 season) at each control. The first survey occurred when most skuas would already have had nests (i.e., in the last decade of December), the second took place somewhere around mid-season, and the third at the end of the season (i.e., in March). If necessary, a single survey was divided into two parts, with the second, complementary inspection occurring as possible after the first, subject to weather conditions.

The frequency of surveys in IBA WAB and the outside regions varied between seasons but generally involved regular controls every 8–12 days for most pairs. Some pairs were monitored every 20 days and, in a few instances, less often, but with no more than a month between surveys.

Species identification was determined on the basis of the visual characteristics, such as plumage coloration, body size, and stature [4,8,30,31]. The diagnostic features for the SPS were a smaller body size than the BS, including a smaller head with flatter crown, narrower wings, slimmer bill (with less pronounced gonys), and plumage features that distinguish between color morphs, from pale (blonde) to dark, through an intermediate.

**Figure 2:**
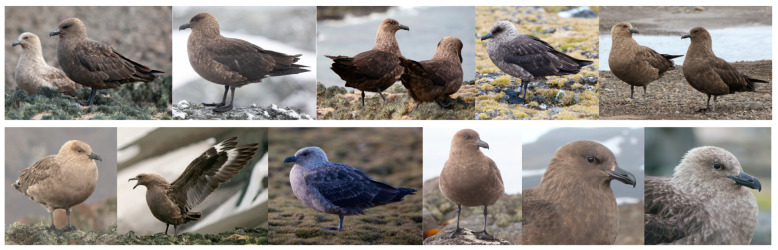
Visual characteristics of SPS with different color morphs.

Diagnostic features of the pale morph adult SPS individuals included a pale head ranging in color from greyish white through light honey to pinkish brown, contrasting with dark brown wing surface and uniformly brown upperparts. The presence of hackles on the neck and nape exhibits a bright sheen, and the underparts are of the same color as the head. Wings are dark brown with characteristic white patches at the base of the primaries. In contrast, the plumage features of the dark morph of adult SPS individuals included head to upperparts dark brown to blackish brown, with the underparts slightly paler. Narrow yellowish-brownish streaks are present on the neck/nape and lighter, whitish on the mantle/scapulars. In the breeding season, visible golden hackles are present on the neck and nape. Intermediate morph between pale and dark is similar to the dark form with lighter barred underparts and hackles on neck with a sheen of intermediate intensity. The color variations described in the text are shown in Figure 2. In all SPS morphs, underwing coverts are dark brown to blackish brown, clearly contrasting with the greyer greater coverts and underside to the flight feathers.

Features in all SPS morphs frequently include, to varying degrees, a light collar at the back of the nape; however, in dark morph, usually faint and, in some cases, limited to a few paler streaks, darker mantle/scapular feathers, a paler band above the base of the bill, and slightly darker coloring of the face/cheeks with darker shadows around and in front of the eyes.

**Figure 3:**
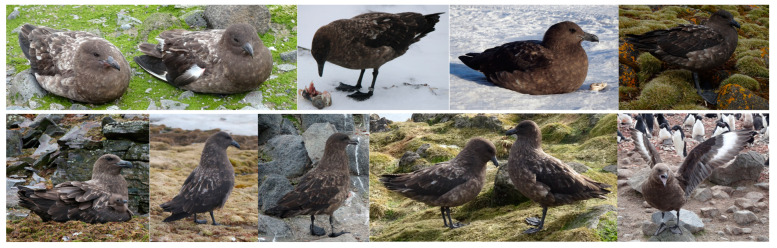
Visual characteristics of BS.

The BS, contrary to the SPS, is larger, heavier and appearing chunkier, with a thicker neck and head that is often topped with a slightly but noticeably darker “cap”, with yellowish streaks on the nape, without the pale collar at the back of the nape, with more patterned dark brown upperparts speckled with yellowish creamy/rufous, similar underparts, but without golden, shiny neck hackles. The upperwing is dark brown with distinct white patches at the base of the primaries, and the underwing is even more patchy. The contrast between lesser and median underwing coverts and greater underwing coverts and underside to flight feathers is less marked than in the SPS. The BS is spotted/streaked more unevenly and broadly than SPS. The characteristics of the BS are presented in Figure 3.

**Figure 4:**
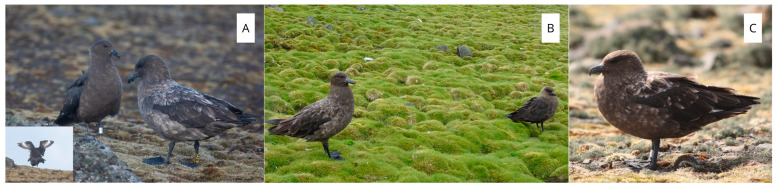
Mixed skua pairs (**A**,**B**) and hybrid individual (**C**).

Individuals with traits intermediate between SPSs and BSs (example shown in Figure 4C, individual with the body size and posture of an SPS and plumage characteristics of a BS), taking into account the differences in size between representatives of the opposite sex in the same species and intraspecific variability, were assigned to the hybrid category; however, the risk of misidentification cannot be excluded, as no genetic analyses were performed. Since only birds with characteristics clearly intermediate between two species were identified as hybrids, it must be assumed that the actual proportion of hybrids in the group studied may be higher than that recorded by distinguishing them based on visual evidence. If a pair consisted of two distinct species (BS and SPS) (example shown in Figure 4A, a pair consisting of a female BS and a male SPS, sex determined by copulation observation, and Figure 4B, a pair formed by an individual of the BS and SPS sex that cannot be determined through observation) or one species (BS or SPS) and a hybrid bird, they were collectively grouped as a “mixed skua pair” (MIX).

The majority of adult skuas on IBA WAB and the outside regions were fitted with leg rings, and the codes on these rings were routinely documented alongside the pair’s territory code. This allowed the data collectors to track any changes in bird composition within pairs or between territories. Skuas from IBA PH also wore leg rings; although, a larger proportion of these birds either did not have a ring or the ring codes remained unread due to the difficulties imposed by the high density of agitated skua pairs. In certain instances, the density of flying and vocalizing birds was too high to determine the actual owner of an encountered nest, resulting in the species being classified as “unknown” (UNK).

Breeding behavior, such as copulation, aggression towards observer/-s, and the presence of eggs or chicks, served as indicators of breeding status. A pair only needed to exhibit one of these behaviors or have offspring present once during the entire season to be considered a “breeding pair”. Non-breeding pairs, which remained in roughly the same location throughout at least part of the season, were labelled as such if they appeared at least twice within a season in the same location without being observed engaging in breeding. It is prudent to note that these pairs might have attempted breeding, but since it was not documented, they were counted as non-breeding pairs. Additionally, these pairs could only be registered if the density of pairs allowed for a conclusive distinction between pairs.

**Figure 5:**
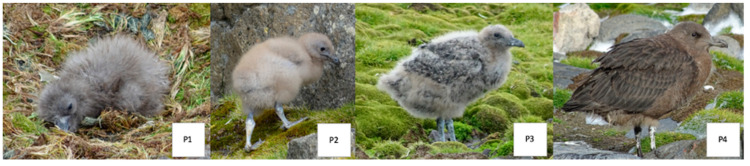
Classification of chick developmental stages (**P1**–**P4**) used in the study.

The developmental stages of chicks were classified as follows: stage “P1” for freshly-hatched chicks remaining in the nest, with only down feathers covering their bodies; stage “P2” for chicks actively exploring the surroundings on visibly long legs, with flight feather buds starting to emerge; stage “P3” for chicks with body feathers covering still visible down feathers, along with long but still growing flight feathers; and stage “P4” for fully fledged chicks capable of flight (Figure 5). If a nest was discovered, the individual territory location was updated to reflect the nest’s location. Furthermore, any recently deceased chicks or their remnants were recorded to differentiate between chicks that likely left the island on their own and those that died before doing so.

In each breeding season and for each surveyed region, the following parameters were documented: the number of breeding and non-breeding pairs, the number of fledged chicks (or an alternative measure if the exact number was unknown), the number of breeding pairs with successful reproduction (including the number of offspring within each pair), and the number of breeding pairs engaged in the breeding process at specific phenological stages.

### 2.3. Discrepancies in Data Used in the Study

The survey area of IBA PH varied between seasons. The 1978/79 [32] and 2022/23 [this study] surveys included Hennequin Point and Smok Hill; whereas, the 2004/05 [5,27] surveys might have included Warkocz [27] or certainly included Warkocz [5]. Warkocz and Smok Hill were also surveyed in the 2023/24 season. However, the survey carried out in the austral summer of 2021/22 [this study] did not include Smok Hill or Warkocz, i.e., it covered only a south-western part of IBA PH, limited in the north to the north-western tip of Dragon Glacier due to weather and logistical constraints. In the case of IBA WAB, data collected between 1977/78 and 2004/05 covered different areas compared to the 2020/21, 2021/22, 2022/23, and 2023/24 seasons. The 1977/78 [33] census focused exclusively on Point Thomas, which is a part of IBA WAB. Subsequent censuses, namely those of 1978/79 [32], 1988/89 [34], 1989/90 [35], 1990/91 [36], 1991/92 [36], 1992/93 [36], 1994/95 [36], and 2004/05 [5], covered a wider area from Point Thomas in the north to Patelnia Point in the south, which coincides with the control area of the current study.

Due to the lower frequency and regularity of inspections in IBA PH compared to IBA WAB, not only flight-capable fledglings (“P4” stage) but also chicks in the “P3” stage observed during the last or penultimate inspection were used as a measure of breeding success. That was due to the fact that the considerable time gap between consecutive inspections did not allow for excluding the possibility that “P3” stage chicks observed at the penultimate inspection were no longer recorded at the last inspection, because they might have gained the ability to fly and moved away from their nesting territory, while the attainment of the “P4” stage by the “P3” chicks observed at the last inspection could not be verified.

As the data presented refer directly to Important Bird and Biodiversity Areas, the species taxonomy used in the paper is consistent with that used by BirdLife International, which places the skua species studied in the genus *Catharacta*.

## 3. Results

**Figure 6:**
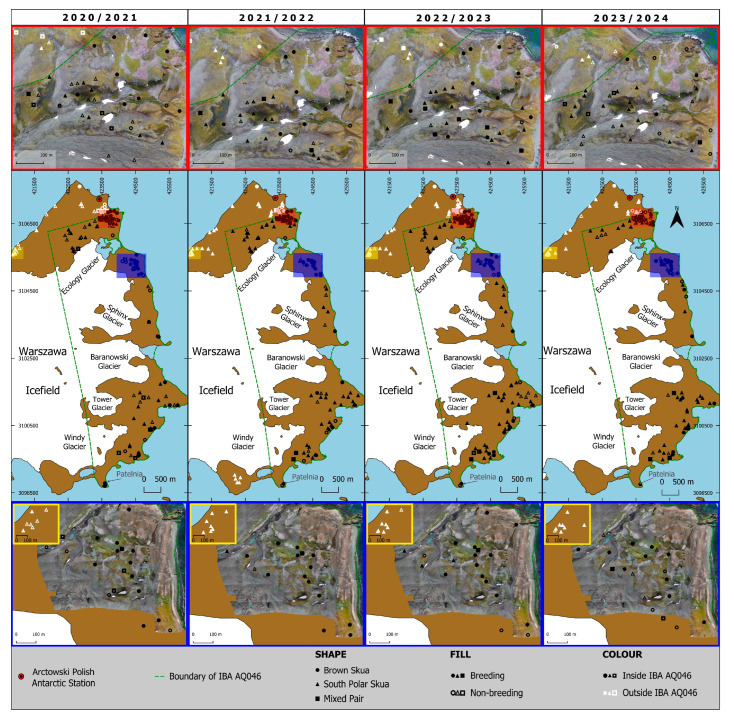
Distribution of skua pairs within and outside IBA WAB boundaries during the four breeding seasons: 2020/21, 2021/22, 2022/23, and 2023/24. The red frames provide close-up views of the area, including Penguin Ridge, Orange Cliff, and partly, Jasnorzewski Garden, where the distribution was densest. The blue frames present close-up views of the Copacabana region, while the yellow frames highlight the Italian Valley area with similarly dense distribution.

**Figure 7:**
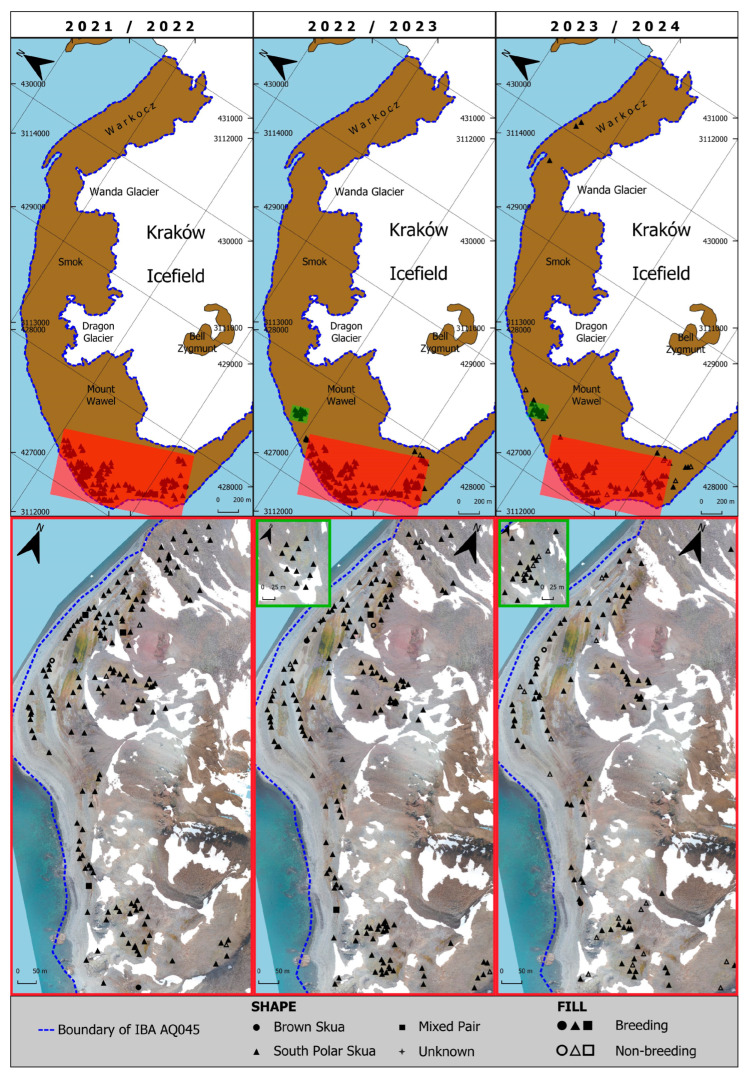
Comparison of the skua pairs distribution within IBA PH during three breeding seasons: 2021/22, 2022/23, and 2023/24. Red and green frames outline regions with the densest recorded distribution.

As demonstrated in Figure 6 and Figure 7, illustrating the spatial distribution of nests and territories within IBA WAB together with its outside regions as well as in IBA PH, respectively, it is evident that there are differences in skua pair distribution in both areas. The areas with the highest pair density have been enlarged for clarity, highlighting the variation in distribution. For IBA WAB, the nests and territories are rather evenly distributed throughout the area; whereas, for IBA PH, they are concentrated mainly in the southern part.

Table 1 and Table 2 present the total number of breeding skua pairs in IBA WAB and IBA PH, respectively. This summary enables a comparison between the currently obtained data and historical data focusing exclusively on breeding pairs. When historical data are considered, the number of breeding pairs observed (mean ± std) for IBA WAB is 75 ± 25, while for IBA PH, excluding 1978/79, it is 141 ± 24.

A comprehensive overview of all skua pairs present in both IBAs is provided in Table 3, which enables an assessment of the global criterion A4 fulfilment for the establishment of an IBA. It is apparent that the SPS meets this condition in both areas; although, it is not the main trigger species for IBA WAB. The average number of total pairs for the investigated seasons (mean ± std) for IBA WAB is 67 ± 7, while for IBA PH, it is 157 ± 18. Conversely, Table 4 offers an exhaustive overview of the total number of nests and territories located in outside regions of IBA WAB, thereby underscoring the fact that a considerable proportion of SPS nests are situated in these areas. Table 5 and Table 6 give a comparison of breeding success for both study areas.

## 4. Discussion

The sympatric zone of brown (BSs) and south polar (SPSs) skuas currently extends for approximately 500 km between 61 and 65° S, covering parts of the Antarctic Peninsula and the South Shetland Islands [9]. The divergent feeding habits exhibited by the two species across their respective ranges imply that their dietary patterns are predominantly influenced by ecological factors rather than taxonomic distinctions [37]. However, in the areas where BSs and SPSs jointly occupy ice-free areas, it is important that these species sufficiently diversify some aspects of their ecological niches to mitigate overall interspecific competition and ensure stable coexistence [10]. This diversity is evident in the choice of nesting sites and foraging patterns. Most studies indicate that BSs tend to nest close to penguin colonies, while SPSs occupy nesting territories located away from these colonies, which, for BSs, prove to be a strategic source of food [3,10,33]. This phenomenon is exemplified by two IBAs, AQ045 (Point Hennequin) and AQ046 (West Admiralty Bay), analyzed in this study. In the IBA PH region, where the presence of penguin colonies has not been documented, a single BS nest was identified among a total of 117–171 SPS nests during the specified period (see Table 2). Conversely, in IBA WAB, together with its outside regions, BS nests have been observed in proximity to the major penguin colonies, predominantly located in the areas of Rakusa Point and Llano Point [38]. In contrast, SPS territories have been noted at a considerable distance from these colonies. This allows BSs to feed on energy-rich eggs and bird meat; whereas, SPSs rely almost exclusively on strictly marine food sources, such as fish, krill, amphipods, and other marine invertebrates [3,10,33,39,40], which have twice the energy quality of bird meat [41].

This feeding niche divergence is attributed to the competitive appropriation of more cost-effective resources by BSs due to their larger body size and aggressiveness [3]. Graña Grilli and Montalti [40] demonstrated coexistence of these species does not always depend on one excluding the other from the use of resources; instead, they can share resources if these are sufficiently abundant. However, the dynamics of shared access to penguin meat in this context might have been changing especially in recent times, as penguin populations in the South Shetland Islands have shown fluctuating trends over the recent four decades. According to Penguindex (pygoscelid penguin-specific biodiversity index, [42]), the abundance of Chinstrap Penguin colonies in the area showed an overall declining trend of 74.4% between 1980 and 2019. It should be noted, nevertheless, that within this time span, upward trends were also recorded (prior to 1989 colonies increased on average by 31.5%). For Adelie Penguin, a comparable population decline of 76.2% was recorded over the same interval, following an initial upward trend. The opposite trend was recorded for the Gentoo Penguin population, with the colony average increasing by 287.6% by 2019.

Studies have indicated that the feeding habits of skuas and the choice of their breeding areas are largely dependent on the availability of penguins combined with the availability of food at sea [4,43,44,45]. In the case of BSs, having a feeding territory in a penguin colony with a relatively predictable food supply can be a strategic advantage during periods of bad weather. Regular provisions of high-quality food should result in the better growth of chicks, as observed in territorial pairs [46], which might translate into their higher survival rates and improved fitness of their parents. According to observations by Trillmich [44], pairs with a foraging territory overlapping with penguin colony start breeding 8–10 days earlier than pairs without a territory and also achieve more eggs and/or chicks per pair. Ritz et al. [47], based on a study conducted on one local SPS subpopulation on King George Island, postulate that the primary factors affecting chick growth in SPS may be the date of hatching, weather factors, and food supply.

In contrast to BSs, sympatric SPSs of the study population foraging at sea [11] do not have the ability to monopolize predictable resources. Moreover, increased competition for fish in larger colonies can lead to an increased foraging range [48,49] and may also cause a depletion of food resources or foraging opportunities [48,49,50,51,52]. According to estimates by Hahn et al. [46], one pair of SPSs rearing two chicks requires about 140 kg of pelagic fish per season. As plunge divers, hunting only in the surface layer of the water, SPSs rely only on fish species that occur in this zone, while competing with other bird or mammal species from the same level in a food web. This competition is likely to intensify because anticipated warming-related decline in krill availability in the Antarctic Peninsula region and the increasing share of gelatinous zooplankton, such as salps in the trophic connections of the high latitudes of the southern hemisphere [53,54], may increase the need to adapt to potential fluctuations in food availability, such as the proportion of pelagic fish in the diets of top predators that previously relied on krill [55,56]. This consideration is the first to suggest a further need to monitor the size of BS and SPS populations, which can provide important information linked to ongoing climate change and the effectiveness of prescribed krill harvest quotas and may be of interest to the Commission for the Conservation of Antarctic Marine Living Resources (CCAMLR).

The last demographic data for the skua population in IBA WAB and IBA PH is from the 2004/05 season [5,27], so the data presented here are an update after a 20-year hiatus. The SPS is a trigger species for IBA PH, and based on the size of its breeding population, the area was classified as an Important Bird and Biodiversity Area in 2015 [20]. The population check we conducted over the three breeding seasons (2021/22, 2022/23, and 2023/24) confirmed that the location still qualifies for IBA status. The species has been classified by IUCN Red List as “least concern”, with a population estimate of 6000–15,000 mature individuals, which means that, in the 2022/23 season in IBA PH, 2.3–5.9% of the pairs of the estimated total population of the species attempted to breed. We observed a 31.6% decrease in the number of SPS breeding pairs between the 2022/23 and 2023/24 seasons in IBA PH and a 37.5% decrease in number of BS breeding pairs in IBA WAB over the same period. However, it is important to highlight that SPS breeding recruitment in IBA PH during the 2022/23 season (171 breeding pairs) was the highest ever reported. The monitoring of BSs and SPSs in Admiralty Bay, and in particular in both investigated IBAs, has been ongoing for almost 50 years [33]; although, it has been intermittent at times [5,27,32,33,34,35,36]. Such data are unique throughout Antarctica and inherently valuable for tracking changes in Antarctic seabird populations, providing a second important justification for the continued monitoring of these two sympatric skua species within Admiralty Bay.

The southern skuas complex can serve as a model system for recently evolved taxa to gain deeper insights into the processes and patterns of early speciation. BSs and SPSs continue to exchange genes in their hybridization zone, and as hypothesized by Ritz et al. [9], population divergence is still incomplete after 200,000 years. Mota et al. [57] largely confirmed Ritz’s theories and posed the following question: How will the evolution of skuas respond to global warming? They also hypothesized that SPSs may not be able to expand their range further north in the face of climate change; whereas, BSs, originating from warmer regions, may be able to expand their range southwards, thus increasing the hybridization zone. While it is important to consider several factors, such as the availability of breeding sites and food supply, these two species are a good model to study, because they are top predators and will reflect the effects of global warming on the Antarctic food web. This seems to be another reason why the monitoring will be crucial in the near future and should be continued in areas where both species of skuas co-occur.

In light of recent information on the presence of confirmed cases of Highly Pathogenic Avian Influenza (HPAI) H5N1, the presented data can be extremely useful in showing the status of skua populations prior to the HPAI virus’ presence in the Antarctic. As migratory birds, partly feeding on carrion and forming their skua clubs in close proximity to freshwater water bodies that may be the virus reservoir, skuas are highly vulnerable to infection. On 8 October 2023, the British Antarctic Survey detected the first case of HPAI clade 2.3.4.4b in a BS on Bird Island, South Georgia [58]. Since then, further cases of HPAI in skuas and other Antarctic bird species have been confirmed [59], but cases involving related skua species from outside Antarctica have also been reported. As the example of the Great Skua population in the Northern Hemisphere shows, populations of this species have been decimated by the epidemic H5N1 HPAI, resulting in mass mortality and local population declines of 60% [60], as estimated from surveillance data from previous seasons.

## 5. Conclusions

The results of the inventories carried out in both IBAs (Point Hennequin, AQ045 and West Admiralty Bay, AQ046) answer the questions posed and allow for the following conclusions to be presented:

(1) The total number of SPS nests in IBA PH in all three seasons (2021/22, 2022/23, and 2023/24) in which the inventory was conducted met the requirement of global criterion A4, exceeding 50 pairs, with 153, 176, and 141 pairs obtained, respectively;
(2) The total number of SPS nests in IBA WAB also exceeded the required 50 pairs in all four seasons (2020/21, 2021/22, 2022/23, and 2023/24), with 63, 66, 77, and 63 recorded, respectively, thus meeting the global criterion A4 for this species. Consequently, it is recommended that IBA WAB be updated and that SPS be incorporated into the criterion as an additional trigger species;
(3) The expansion of both IBA boundaries is recommended.
  (a) IBA PH, as the exposure of new ice-free areas, has the potential to host new SPS and BS nests;
  (b) In the case of IBA WAB, 24 ± 3 SPS nests have been found outside the IBA boundaries, which on their own cannot form a separate IBA but could complement the existing one, particularly in the areas of Jasnorzewski Garden and Italian Valley.

## Figures and Tables

**Table 1:** Number of breeding pairs of skuas recorded during various breeding seasons in IBA WAB.

Species	1977/78 ^1^	1978/79 ^2^	1988/89 ^3^	1989/90 ^4^	1990/91 ^5^	1991/92 ^5^	1992/93 ^5^	1994/95 ^5^	2004/05 ^6^	2020/21	2021/22	2022/23	2023/24
BS	at least 28	47							42	29	28	32	20
SPS	at least 13	12							72	24	58	64	47
MIX	at least 4	10							16	4	7	9	7
Total	at least 45	69	38	57	62	71	75	64	130	57	93	105	74

^1^ Trivelpiece et al. [33]; ^2^ Jabłoński [32]; ^3^ Sierakowski [34]; ^4^ Lesiński [35]; ^5^ Sierakowski [36]; ^6^ Carneiro et al. [5].

**Table 2:** Number of breeding pairs of skuas recorded during various breeding seasons in IBA PH.

Census Period						
Species	1978/79 ^1^	2004/05 ^2^	2004/05 ^3^	2021/22	2022/23	2023/24
BS	12	2	1	1	1	1
SPS	10	116	113	149	171	117
MIX	0	8	15	3	2	0
UNK	-	-	-	3	1	0
Total	22	126	129	156	175	118

^1^ Jabłoński [32]; ^2^ Costa and Alves [27]; ^3^ Carneiro et al. [5].

**Table 3:** Number of breeding (BR) and non-breeding (NBR) pairs of skua species (Sp.) on IBA WAB and IBA PH in the seasons 2020/21, 2021/22, 2022/23, and 2023/24.

		Census Period											
		2020/2021			2021/2022			2022/2023			2023/2024		
**Sp.**	**IBA**	**BR**	**NBR**	**Total Pairs**	**BR**	**NBR**	**Total Pairs**	**BR**	**NBR**	**Total Pairs**	**BR**	**NBR**	**Total Pairs**
BS	WAB	29	13	42	28	7	35	32	8	40	20	10	30
	PH	-	-	-	1	1	2	1	1	2	1	2	3
SPS	WAB	24	39	63	58	8	66	64	13	77	47	16	63
	PH	-	-	-	149	4	153	171	5	176	117	24	141
MIX	WAB	4	12	16	7	2	9	9	2	11	7	3	10
	PH	-	-	-	3	0	3	2	0	2	0	0	0
UNK	PH	-	-	-	3	0	3	1	0	1	0	0	0

**Table 4:** Number of breeding (BR) and non-breeding (NBR) pairs of skuas in the outside regions of IBA WAB. The abbreviation ‘N’ refers to the areas located to the north and northwest, while ‘S’ refers to the areas situated to the southwest of IBA WAB.

		Census Period							
		2020/2021		2021/2022		2022/2023		2023/2024	
Species	Location	BR	NBR	BR	NBR	BR	NBR	BR	NBR
BS	N	1	3	2	3	4	0	1	1
	S	-	-	0	0	-	-	-	-
SPS	N	7	12	24	0	25	2	21	5
	S	-	-	5	0	-	-	-	-
MIX	N	0	3	1	0	2	0	0	0
	S	-	-	0	0	-	-	-	-

**Table 5:** Breeding success of skuas in IBA WAB in the seasons 2020/21, 2021/22, 2022/23, and 2023/24 presented as a number of pairs with one or two fledglings that have acquired the ability to fly.

Census Period	Species	Number of Pairs with Two Fledglings	Number of Pairs with One Fledgling	Number of Fledglings
2020/2021	BS	3	15	21
	SPS	0	0	0
	MIX	0	0	0
2021/2022	BS	8	8	24
	SPS	0	7	7
	MIX	0	2	2
2022/2023	BS	2	13	17
	SPS	0	26	26
	MIX	0	2	2
2023/2024	BS	4	9	17
	SPS	0	0	0
	MIX	0	0	0

**Table 6:** Breeding success of skuas in IBA PH during the seasons 2021/22, 2022/23, and the 2023/24 season presented as a number of pairs with different numbers of offspring in two stages of juvenile plumage development.

Census Period	Species	Number of Pairs with Two Fledglings	Number of Pairs with One Fledgling	Number of Pairs with Two Chicks	Number of Pairs with One Chick	Number of Pairs with at Least One Chick or Fledgling
2021/2022	BS	0	0	0	0	0
	SPS	0	30	1	9	40
	MIX	0	0	0	0	0
	UNK	0	0	0	0	0
2022/2023	BS	0	1	0	0	1
	SPS	2	30	1	5	38
	MIX	0	1	0	0	1
	UNK	0	0	0	0	0
2023/2024	BS	0	0	0	0	0
	SPS	0	0	0	1	1

## Data Availability

Dataset available on request from the authors.

## Appendix A

**Table A1:** Timing of each stage during the 2020/2021, 2021/2022, 2022/2023, and 2023/2024 breeding seasons of Antarctic skuas on the West Shore of Admiralty Bay (IBA AQ046).

		Species											
		BS				SPS				MIXED			
	Census Period	2020/2021	2021/2022	2022/2023	2023/2024	2020/2021	2021/2022	2022/2023	2023/2024	2020/2021	2021/2022	2022/2023	2023/2024
Stage of the Breeding Season *													
1		31.10–20.11	23.10–09.11	03.10–04.11	22.10–14.11	22.11–12.12	18.11–05.12	17.11–14.12	24.11–10.12	10.11–04.01	15.11–07.12	05.11–06.12	06.11–01.12
2		21.11–23.12	10.11–19.12	05.11–09.12	15.11–22.12	13.12–16.02	06.12–24.01	15.12–26.01	11.12–08.01	05.01–25.01	08.12–11.01	07.12–04.01	02.12–16.01
3		24.12–22.01	20.12–24.01	10.12–04.01	23.12–27.01	17.02–09.03	25.01–31.01	27.01–10.02	09.01–28.01	26.01–26.02	12.01–09.02	05.01–27.01	17.01–28.01
4		23.01–18.02	25.01–16.02	05.01–10.02	28.01–10.02	10.03–26.03	01.02–16.03	11.02–02.03	N/A	N/A	10.02–16.03	28.01–08.03	N/A
5		19.02–13.04	17.02–31.03	11.02–29.03	11.02–26.03	27.03–31.03	17.03–30.04	03.03–29.03	N/A	N/A	17.03–31.03	09.03–29.03	N/A

* The numbers used in the table represent the following stages: “1”—arrival of adults and establishment of territories, “2”—nest building, egg/s laying, and incubation; “3”—presence of young chick/s (stage “P1”–“P2”); “4”—presence of older chick/s (stage “P3”–“P4”); “5”—presence of fully fledged chick/s capable of flight and their departure.

**Table A2:** Number of breeding pairs of Antarctic skuas observed actively engaging in breeding process, including copulation, incubation of eggs, protection, feeding, and tending chicks, across five phenological stages during the breeding seasons of 2020/21, 2021/22, 2022/23, and 2023/24 on the West Shore of Admiralty Bay (IBA AQ046).

		Species											
		BS				SPS				MIXED			
	Census Period	2020/2021	2021/2022	2022/2023	2023/2024	2020/2021	2021/2022	2022/2023	2023/2024	2020/2021	2021/2022	2022/2023	2023/2024
Stage of the Breeding Season													
1		8	2	9	10	2	4	33	34	0	0	4	2
2		26	26	24	18	21	56	58	46	3	6	8	7
3		23	22	20	16	3	37	45	12	3	5	6	4
4		22	19	18	14	0	15	38	0	0	3	2	0
5		18	16	15	13	0	7	26	0	0	2	2	0

**Table A3:** Number of breeding pairs of Antarctic skuas that was observed actively engaging in breeding processes, i.e., displaying highly territorial behaviors, incubating eggs, protecting, feeding, or tending chicks across three phenological stages of the 2020/21, 2021/22, and 2022/23 breeding seasons in southern part of Point Hennequin (IBA AQ045) and within the entire area of the IBA AQ045 during the 2023/24 season.

	Census Period									
	2021/2022				2022/2023				2023/2024	
Species Timespan	BS	SPS	MIXED	UNK	BS	SPS	MIXED	UNK	BS	SPS
21.12–04.02	1	139	2	2	2	176	2	1	2	117
05.02–05.03	0	50	1	1	1	42	1	0	0	25
06.03–03.04	0	33	0	0	1	32	1	0	0	0
